# *COMT* and *MAO-A* Polymorphisms and Obsessive-Compulsive Disorder: A Family-Based Association Study

**DOI:** 10.1371/journal.pone.0119592

**Published:** 2015-03-20

**Authors:** Aline Santos Sampaio, Ana Gabriela Hounie, Kátia Petribú, Carolina Cappi, Ivanil Morais, Homero Vallada, Maria Conceição do Rosário, S. Evelyn Stewart, Jesen Fargeness, Carol Mathews, Paul Arnold, Gregory L. Hanna, Margaret Richter, James Kennedy, Leonardo Fontenelle, Carlos Alberto de Bragança Pereira, David L. Pauls, Eurípedes Constantino Miguel

**Affiliations:** 1 Department and Institute of Psychiatry, Faculdade de Medicina da Universidade de São Paulo, São Paulo, SP, Brazil; 2 Serviço Médico Universitário, Universidade Federal da Bahia (UFBA, Federal University of Bahia), Salvador, BA, Brazil; 3 Universidade de Pernambuco (UPE, University of Pernambuco) Faculdade de Ciências Médicas, Recife, PE, Brazil; 4 Universidade Federal de Sao Paulo (UNIFESP, Federal University of São Paulo), São Paulo, SP, Brazil; 5 British Columbia Mental Health and Addictions Research Institute, Vancouver, BC, Canada; 6 Psychiatric and Neurodevelopmental Genetics Unit (PNGU), Massachusetts General Hospital, Boston, Massachusetts, United States of America; 7 Center for Human Genetics Research, Massachusetts General Hospital, Boston, Massachusetts, United States of America; 8 Department of Psychiatry, University of California, San Francisco, California, United States of America; 9 Program in Genetics and Genome Biology, The Hospital for Sick Children, Toronto, Ontario, Canada; 10 Department of Psychiatry, University of Michigan, Ann Arbor, Michigan, United States of America; 11 The Frederick W. Thompson Anxiety Disorders Centre, Sunnybrook Health Sciences Centre, Toronto, Ontario, Canada; 12 Centre for Addiction and Mental Health, University of Toronto, Toronto, Ontario, Canada; 13 Programa de Ansiedade e Depressão, Instituto de Psiquiatria, Universidade Federal do Rio de Janeiro (IPUB/UFRJ), Rio de Janeiro, RJ, Brazil; 14 Department of Statistics, The Institute of Mathematics and Statistics, University of São Paulo, SP, Brazil; Vanderbilt University, UNITED STATES

## Abstract

**Objective:**

Obsessive-compulsive disorder (OCD) is a common and debilitating psychiatric illness. Although a genetic component contributes to its etiology, no single gene or mechanism has been identified to the OCD susceptibility. The catechol-O-methyltransferase (COMT) and monoamine oxidase A (MAO-A) genes have been investigated in previous OCD studies, but the results are still unclear. More recently, Taylor (2013) in a comprehensive meta-analysis of genetic association studies has identified *COMT* and *MAO-A* polymorphisms involved with OCD. In an effort to clarify the role of these two genes in OCD vulnerability, a family-based association investigation was performed as an alternative strategy to the classical case-control design.

**Methods:**

Transmission disequilibrium analyses were performed after genotyping 13 single-nucleotide polymorphisms (eight in *COMT* and five in *MAO-A*) in 783 OCD trios (probands and their parents). Four different OCD phenotypes (from narrow to broad OCD definitions) and a SNP x SNP epistasis were also analyzed.

**Results:**

OCD, broad and narrow phenotypes,were not associated with any of the investigated *COMT* and *MAO-A* polymorphisms. In addition, the analyses of gene-gene interaction did not show significant epistatic influences on phenotype between *COMT* and *MAO-A*.

**Conclusions:**

The findings do not support an association between DSM-IV OCD and the variants of *COMT* or *MAO-A*. However, results from this study cannot exclude the contribution of these genes in the manifestation of OCD. The evaluation of broader spectrum phenotypes could help to understand the role of these and other genes in the pathophysiology of OCD and its spectrum disorders.

## Introduction

Obsessive-compulsive disorder (OCD) is characterized by repetitive thoughts (obsessions) and repetitive behaviors (compulsions) that are unwanted, time consuming, egodystonic, and result in significant functional impairment [1]. Various studies have demonstrated that the etiology of OCD has a genetic component [2] with reported heritability rates ranging between 27% and 65% [3]. Although more than 140 candidate gene studies have been conducted, the findings have been inconclusive due to small sample size and few study replications [4]. The etiology of OCD seems to involve an interaction between environmental factors and several genes of small effect [2]. Studies employing different methodological strategies (e.g., cerebral spinal fluid metabolites measurements, pharmacological challenges, and drug treatment responses) all suggest that abnormal serotonergic neurotransmission is one of the most consistent biological findings in OCD [5]. Studies have also reported dopaminergic abnormalities in the basal ganglia and nucleus accumbens, as well as altered glutamate transmission in other locations in OCD samples, thus suggesting that complex interactions between multiple neurotransmitter systems may contribute to the phenotypic presentation of OCD [6]. This could be understood by a potential dysfunction in enzymes that metabolize central nervous system neurotransmitters in OCD. Two important enzymes of this type are catechol-*O*-methyltransferase (COMT) and monoamine oxidase A (MAO-A).

COMT is an Mg^2+^-dependent enzyme involved in the inactivation of certain catecholamines (norepinephrine, epinephrine, and dopamine). The important role that COMT plays in neuropsychiatric disorders has previously been described [7]. The most widely studied *COMT* polymorphism is a single-nucleotide polymorphism (SNP) leading to a valine-to-methionine substitution at codon 158 (val^158^met or rs4680), which results in a low-activity thermolabile and a high-activity thermostable forms of the enzyme—the met^(158)^ and val^(158)^ alleles, respectively [8].

A deletion within the 22q11 region, which includes the *COMT* region, causes velocardiofacial syndrome, which increases the risk for many psychiatric disorders [9], including OCD [10]. Although various studies have evaluated the association between *COMT* polymorphisms and OCD, the findings have been inconclusive [4].

The monoamine oxidases (MAO) are enzymes that catalyze the oxidation of monoamines, which exist in two forms: MAO-A and MAO-B. Both forms are found bound to the outer membrane of mitochondria in most cell types in the body but show different specificities. MAO-A is present in catecholaminergic neurons in the brain and plays a preferencial role in the metabolic degradation of several neurotransmitters, including serotonin, norepinephrine, epinephrine, and dopamine. The *MAO-A* gene maps to the p11.3 region on the X chromosome. The first evidence that *MAO-A* could play an important role in human behavior was the description of a large Dutch family that presented with a new form of mental retardation with prominent behavioral abnormalities linked to the X chromosome. Several males in the family were affected by this disturbance and exhibited aggressive behavior, with significant impulsivity, pyromania, suicide attempts, and sexually aberrant behavior [11,12].

Some studies that have investigated the association between *MAO-A* polymorphisms and OCD, and they have produced controversial results [4]. The various methodologies, different phenotype definitions, small sample sizes and diverse ethnic backgrounds of the populations studied could be sources of bias contributing to the discrepant results obtained in previous studies.

Recently, Taylor [13] performed a comprehensive meta-analysis of OCD genetic association studies. A total of 230 polymorphisms from 113 genetic association studies were identified. In the main meta-analysis, *COMT* and *MAO-A* polymorphisms were found to be associated with OCD in males.

In this study, the transmission disequilibrium of *COMT* and *MAO-A* SNPs was analyzed in OCD patients, using a family-based association approach. Given that family studies have shown familial aggregation between OCD and different psychiatric disorders (e.g., tic disorders, body dysmorphic disorder, skin picking, trichotillomania and anxiety disorders) [14,15,16,17,18,19], these disorders were included as broader phenotypes in the analyses.

## Methods

### Participants

The sample consisted of 783 individuals meeting DSM-IV criteria for OCD, and their biological parents. The OCD probands were recruited from specialized OCD clinics at Harvard University, the University of California- San Francisco, the University of Michigan, the University of Toronto, as well as from two universities in Brazil—the University of São Paulo and the University of Pernambuco that participate from the Obsessive-Compulsive Foundation Genetics Collaborative Subgroup. Most of the sample was studied in a genome-wide association study [20]. The probands and their parents underwent structured clinical interviews. For subjects who were ≥ 16 years of age, we employed the Structured Clinical Interview for DSM-IV Axis I Disorders, Clinician Version [21]. For subjects who were < 16 years of age, we employed the Schedule for Affective Disorders and Schizophrenia for School Aged Children—Present and Lifetime Version (K-SADS-PL) [22]. In addition, all subjects were interviewed with the Yale-Brown Obsessive Compulsive Scale [23] and the Yale Global Tic Severity Scale [24]. All of the interviewers were trained mental health professionals (psychiatrists or psychologists). Reliability evaluations were performed and best-estimate diagnoses were made. The assessment procedures have previously been described in detail [25].

### Ethics Statement

This work was performed in accordance with Declaration of Helsinki. The project was approved by the research ethics committees (IRB) at each of the participating institutions (In Pernambuco, Brazil: Complexo Hospitalar do Hospital Universitario Oswaldo Cruz e Pronto Socorro Cardiologico de Pernambuco, University of Pernambuco – HUOC/PROCAP #29/2010; and In Sao Paulo, Brazil: Comissao de Etica para Analise de Projetos de Pesquisa do Instituto de Psiquiatria da Faculdade de Medicina da Universidade de Sao Paulo—CAPPesq IPqHCFMUSP #968/05; United States: University of California, San Francisco Committee on Human Research—#10–00157; Institutional Review of the University of Michigan Medical School (IRBMED) protocol #1992-0191; Canada: Toronto: Centre for Addiction and Mental Health Research Ethics Board #368-2008). Written informed consent was obtained from all adult participants and from the parents of those who were minors.

### Narrow and Broad Phenotypes categories

In addition to the association analysis between *COMT* and *MAO-A* and DSM-IV OCD, additional analyses were performed in the Brazilian subsample (83 trios) using the broader phenotypes described below.

Hypothesizing that OCD phenomenology (and not severity) could be influenced by COMT and MAO-A genes, we examined the association of COMT and MAO-A polymorphisms and a broadly-defined OCD, which includes cases with OCD symptoms even when they did not meet full DSM-IV criteria for OCD (subclinical OCD).Acknowledging that OCD is part of a spectrum [26,27] that includes other anxiety disorders [19,28], body dysmorphic disorder, and pathological grooming disorders (skin picking and trichotillomania) [17], these conditions were collectively referred to as OCD spectrum disorders. The term ‘OCD spectrum disorders’ includes DSM-IV OCD, subclinical OCD, together with the DSM-IV anxiety disorders, body dysmorphic disorder, and pathological grooming disorders (skin picking and trichotillomania).

In addition, since some studies have suggested that OCD could be grouped based on tic disorder comorbidity [29,30], analyses in the Brazilian subsample were also completed to examine an association between *COMT* and *MAO-A* polymorphisms and OCD with tic disorder (Tourette syndrome or chronic tic disorder) comorbidity.

### Genotyping

DNA from peripheral blood samples were extracted using the “salting-out” protocol [31]. On the basis of Tagger software [32], *COMT* and *MAOA* genomic regions were specified and tag SNPs were picked (a local copy of HapMap data was used) within them. Tagger produced a list of tag SNPs and corresponding statistical tests to capture all variants of interest, and a summary coverage report of the selected tag SNPs. Eleven SNPs in the *COMT* gene region and six SNPs in the *MAO-A* gene region were selected. These SNPs were genotyped at the Psychiatric and Neurodevelopmental Genetics Unit of Massachusetts General Hospital, a teaching hospital associated with Harvard University. Genotyping was performed in 384-well plates with the Sequenom MassARRAY platform (Sequenom, San Diego, CA, USA). Primers for polymerase chain reaction (PCR) amplification and single-base extension assays were designed using Assay Design software, version 3.1 on the basis of FASTA sequences surrounding the SNPs, derived from SNPper [33]. Multiplex PCR was performed, followed by a pooled single-base extension reaction (iPLEX Gold). Samples were analyzed with a MassARRAY RT mass spectrometer, in automated mode. The resulting spectra were analyzed with SpectroTyper software after baseline correction and peak identification (Sequenom). Information about the ten *COMT* SNPs and the six *MAO-A* SNPs was obtained from the dbSNP, Celera, and HapMap databases. Three markers were excluded because of quality control issues: *COMT* rs2239393, because the genotyping success rate was lower than 75%; *MAO-A* rs2179098, because the minor allele frequency was ≤ 0.025; and *COMT* rs1544325, because the Hardy-Weinberg p value was < 0.05. The thirteen remaining SNPs collectively covered the *COMT* and *MAO-A* gene regions: the eight selected *COMT* SNPs spanned 25.6 kb, with a density of 2.84 kb/SNP; and the five selected *MAO-A* SNPs spanned 63 kb, with a density of 12.6 kb/SNP. Single-marker and haplotype analyses were performed in order to identify associations.

### Statistical analysis

Statistical analyses were performed with the programs PLINK [34,35] and Haploview 3.32 [36]. Using the Haploview software, SNPs were selected on the basis of quality control criteria. 95% confidence bounds on D prime are generated and each comparison is called "strong LD", "inconclusive" or "strong recombination". A haplotype block is created if 95% of informative (i.e., non-inconclusive) comparisons are "strong LD" [37]. Following this, the haplotype TDT analysis was performed.

For single markers association, the results of the standard transmission/disequilibrium test (TDT) and the `parental discordance test` [34] were combined in order to calculate the p-value using PLINK [34,35]. No covariate was added in the TDT analysis model. The `parental discordance test`is based on a comparison between affected and unaffected parents in terms of the number of alleles they carry, treating each parental pair as a matched case-control pair. The `parental discordance test` assumes homogeneity, in terms of population stratification, within rather than between nuclear families. This test can add power to family-based association analyses, as well as provide considerable protection against population stratification [38].

In addition to the association analysis between *COMT* and *MAO-A* and DSM-IV OCD, a secondary investigation of the *COMT* and *MAO-A* associations in our sample was completed. First, probands were grouped into gender-matched trios in order to evaluate the influence of gender on the results. This approach was based on the knowledge that COMT has influence on estrogens [39] and that *MAO-A* is located on the X chromosome.

Second, exploratory analyses were performed on the Brazilian subsample to consider as affected status, some OCD-related phenotypes such as broadly-defined OCD (clinical plus subclinical OCD), OCD spectrum disorders and OCD with comorbid tic disorders.

Third, since both *COMT* and *MAOA* are involved in dopamine catabolism, the gene- gene interaction was evaluated in contributing to OCD etiology. It was used as the case-only epistatic analysis under the SNP x SNP model, using PLINK software [34,35] in the whole sample (783 trios). Only SNPs that are more than 1 Mb apart, or on different chromosomes, are included in case-only analyses.

The statistical power of this study was calculated using Genetic Power Calculator [40].

The results were subject to permutation analysis (100,000 permutations) using Plink, in order to control for false-positive association, and to Bonferroni`s correction for multiple analysis (corrected p-value threshold = 0.0004).

## Results

### Strictly-defined DSM-IV OCD phenotype

The results of the OCD association analysis of the eight *COMT* SNPs and five *MAO-A* SNPs are described in Table 1. The TDT, the “parental discordance test”, and the combined test were all not significant for the female (Table 2) and the male proband trios (Table 3).

**Table 1:** Single-marker analysis of OCD association with catechol-*O*-methyltransferase and monoamine oxidase A single-nucleotide polymorphisms.

Gene	SNP	OR	CHISQ	P	CHISQ_PAR	P_PAR	CHISQ_COM	P_COM
***COMT***	rs737866	0.756	6.127	0.013	2.286	0.131	7.86	0.005
	rs933271	1.238	3.191	0.074	0.222	0.637	3.413	0.065
	rs5993883	1.164	2.042	0.153	1.882	0.17	3.133	0.077
	rs740603	0.97	0.091	0.762	2.574	0.109	0.057	0.812
	rs4680	1.034	0.099	0.752	1	0.317	0.002	0.96
	rs4646316	0.851	1.766	0.184	0.143	0.706	1.325	0.25
	rs165774	0.988	0.012	0.911	0.037	0.847	0.026	0.872
	rs9332377	0.88	0.832	0.362	0.6	0.439	1.174	0.278
***MAO-A***	rs1465107	0.957	0.066	0.798	0.4	0.527	0.277	0.599
	rs1465108	1.016	0.008	0.929	0.026	0.873	0	1
	rs6323	1.014	0.007	0.934	1.4	0.237	0.2	0.655
	rs979606	1.094	0.269	0.604	0.471	0.493	0.024	0.877
	rs979605	1.103	0.343	0.558	0.641	0.423	0.022	0.882

SNP: single-nucleotide polymorphism; TDT: transmission/disequilibrium test; OR: TDT odds ratio; CHISQ: TDT chi-square value; P: TDT p value; CHISQ_PAR: parental discordance test chi-square value; P_PAR: parental discordance test p value; CHISQ_COM: combined test chi-square value; P_COM: combined test p value; *COMT*: catechol-*O*-methyltransferase; *MAO-A*: monoamine oxidase-A

**Table 2:** Analysis of OCD association with catechol-*O*-methyltransferase and monoamine oxidase A single-nucleotide polymorphisms in female proband trios.

Gene	SNP	OR	CHISQ	P	CHISQ_PAR	P_PAR	CHISQ_COM	P_COM
***COMT***	rs737866	0.649	5.541	0.019	0.4	0.527	5.939	0.015
	rs933271	1.137	0.449	0.503	0.667	0.414	0.217	0.641
	rs5993883	1.463	4.699	0.03	2.579	0.108	6.737	0.009
	rs740603	1.295	2.314	0.128	4.167	0.041	4.78	0.029
	rs4680	1.293	2.173	0.14	0.4	0.527	1.573	0.21
	rs4646316	0.893	0.34	0.56	0	1	0.3	0.585
	rs165774	0.875	0.533	0.465	1	0.317	0.194	0.66
	rs9332377	0.783	1.22	0.269	0.111	0.739	0.89	0.345
***MAO-A***	rs1465107	1.083	0.08	0.777	0.067	0.796	0.015	0.901
	rs1465108	1.087	0.083	0.773	0.067	0.796	0.016	0.9
	rs6323	1.24	0.643	0.423	0.692	0.405	0.13	0.718
	rs979606	1.2	0.455	0.5	0.692	0.405	0.059	0.808
	rs979605	1.269	0.83	0.362	0.692	0.405	0.222	0.637

SNP: single-nucleotide polymorphism; TDT: transmission/disequilibrium test; OR: TDT odds ratio; CHISQ: TDT chi-square value; P: TDT p value; CHISQ_PAR: parental discordance test chi-square value; P_PAR: parental discordance test p value; CHISQ_COM: combined test chi-square value; P_COM: combined test p value; *COMT*: catechol-*O*-methyltransferase; *MAO-A*: monoamine oxidase-A

**Table 3:** Analysis of OCD association with catechol-*O*-methyltransferase and monoamine oxidase A single-nucleotide polymorphisms in male proband trios.

Gene	SNP	OR	CHISQ	P	CHISQ_PAR	P_PAR	CHISQ_COM	P_COM
***COMT***	rs737866	0.827	1.705	0.192	0.889	0.346	2.327	0.127
	rs933271	1.28	2.579	0.108	0.111	0.739	2.689	0.101
	rs5993883	1.037	0.073	0.786	0.067	0.796	0.107	0.743
	rs740603	0.851	1.613	0.204	0.043	0.835	1.332	0.248
	rs4680	0.955	0.114	0.736	1	0.317	0.345	0.557
	rs4646316	1.099	0.424	0.515	0.889	0.346	0.12	0.73
	rs165774	0.918	0.214	0.644	2.667	0.102	0.658	0.417
	rs9332377	0.954	0.048	0.827	0	1	0.037	0.847
***MAO-A***	rs1465107	1.054	0.053	0.818	0.037	0.847	0.087	0.768
	rs1465108	0.954	0.046	0.829	0.222	0.637	0.154	0.695
	rs6323	1.111	0.21	0.646	0.048	0.827	0.094	0.761
	rs979606	1.077	0.111	0.739	0	1	0.087	0.768
	rs979605	0.827	1.705	0.192	0.889	0.346	2.33	0.127

SNP: single-nucleotide polymorphism; TDT: transmission/disequilibrium test; OR: TDT odds ratio; CHISQ: TDT chi-square value; P: TDT p value; CHISQ_PAR: parental discordance test chi-square value; P_PAR: parental discordance test p value; CHISQ_COM: combined test chi-square value; P_COM: combined test p value; *COMT*: catechol-*O*-methyltransferase; *MAO-A*: monoamine oxidase-A

### Haplotypes

**Fig 1:**
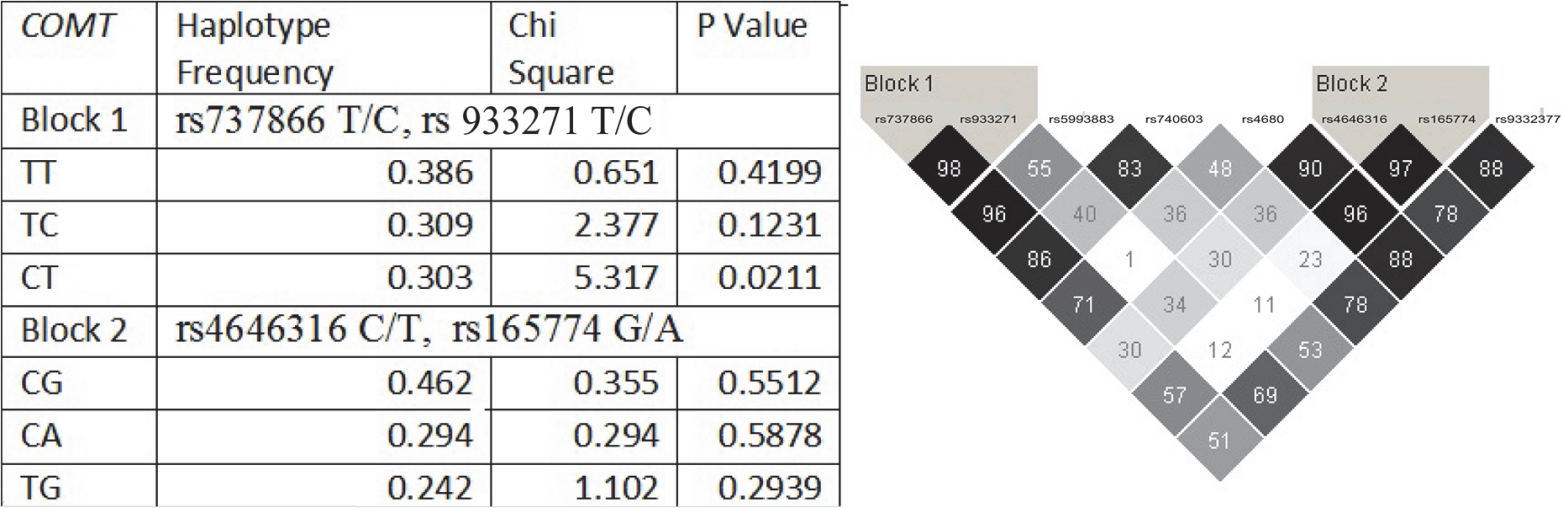
Catechol-*O*-methyltransferase haplotype blocks composed by single nucleotide polymorphisms evaluated. Two haplotype blocks, with two SNPs each, were defined in Cathecol-*O*-methyltransferase. Block 1 comprises SNP rs737866 and rs933271 and Block 2 comprises SNPs rs4646316 and rs165774. Darker squares mean the high D prime and high LOD scores. Haplotype blocks were defined with D prime scores higher than 95. When D prime is 100, the number is not shown inside the square. Two haplotype blocks, with two SNPs each, were defined in Cathecol-*O*-methyltransferase. Data shown in the table refers to each haplotypes allele combination association results.

**Fig 2:**
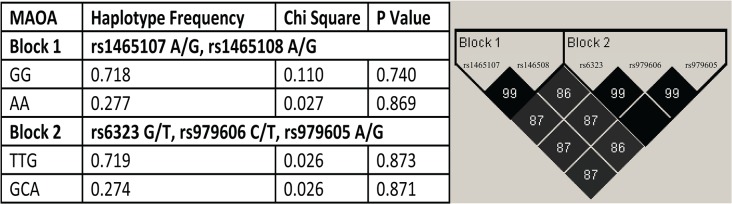
Monoamine oxidase A haplotype Block comprises SNPs rs1465107 and rs1465108 and Block 2 comprises the SNPs rs6323, rs979606, and rs979605. 1 composed by single nucleotide polymorphisms evaluated. Darker squares mean the high D prime and high LOD scores. Haplotype blocks were defined with D prime scores higher than 95. When D prime is 100, the number is not shown inside the square. Data shown in the table refers to each haplotypes allele combination association results.

Two haplotype blocks were identified in each gene (Figs. 1 and 2). In the *COMT* gene (Fig. 1), SNPs rs737866 and rs933271 compose block 1; and SNPs rs4646316 and rs165774 compose block 2. In the *MAO-A* gene (Fig. 2), SNPs rs1465107 and rs1465108 compose block 1; and the SNPs rs6323, rs979606, and rs979605 compose block 2. None of these haplotype blocks were found to be associated with OCD (Figs. 1 and 2).

### Epistasis analysis

None of the *COMT* or *MAOA* SNPs showed an epistatic interaction in association with OCD (data not shown).

### Power calculation for whole sample

The statistical power for this association analysis (783 trios, high risk allele frequency = 0.478; OCD prevalence = 0.025; alpha = 0.05) was 65% in average for *COMT* gene (1120 trios were needed to achieve 80% power) and 58% in average for *MAOA* (783 trios, high risk allele frequency = 0.257; OCD prevalence = 0.025; alpha = 0.05; and 1305 trios needed to achieve 80% power).

A secondary analysis was performed, with the Brazilian subsample, as described below:

### Strictly-defined OCD with comorbid tic disorders

When the Brazilian patients with OCD and comorbid tic disorders (Tourette syndrome or chronic tic disorders; N = 19 trios) were analyzed separately, OCD with comorbid tic disorder was not found to be significantly associated with any *COMT* or *MAO-A* SNP (S1 Table).

### Broadly-defined OCD phenotype

When the Brazilian subjects meeting criteria for subclinical and clinical OCD are included together, there was preferential transmission of the A allele of the *MAO-A* SNP rs1465108 (p = 0.0469) (S2 Table). This finding did not remain significant after 100,000 permutations nor after Bonferroni`s correction.

### Broadly-defined OCD spectrum disorders

Subsequent analyses revealed that the A allele of the *MAO-A* SNP rs979605 was associated with OCD spectrum disorders (as defined above: including patients with OCD, subclinical OCD, skin picking, trichotillomania, body dysmorphic disorder, or DSM-IV-defined anxiety disorders) (p = 0.027) (S3 Table). This finding also did not remain significant after 100,000 permutations nor after Bonferroni`s correction.

### Power calculation for Brazilian subsample

The statistical power for the analyses performed with the Brazilian subsample (83 trios; high risk allele frequency = 0.39; OCD prevalence = 0.025; alpha = 0.05) was 11% (1282 trios needed for 80% power) for *COMT;* and for *MAOA* would be 10.5% power (83 trios; high risk allele frequency = 0.32; OCD prevalence = 0.025; alpha = 0.05; and 1402 trios needed for 80% power)

## Discussion

The results presented here do not support the hypothesis that DSM-IV OCD diagnosis is associated with *MAO-A* or *COMT*. Results were also negative in the additional analyses performed: 1) one that examined a strictly defined phenotype that consisted of individuals with OCD and tic disorders; 2) one that examined a broadly defined phenotype that included OCD, subclinical OCD; 3) one that included other OCD spectrum disorders; and 4) one *COMT-MAOA* epistasis analysis.

This result is consistent with the findings of a previous Brazilian study where no association between DSM-IV OCD and the *COMT* val-158-met (rs4680) variant in a case-control OCD study was observed (Meira-Lima et al. 2004).

As with any genetic association study, the present work is not without its limitations. The main limitation is the small sample size. The statistical power to refute the null hypothesis in this study was 23% (*COMT*) and 21% (*MAOA*) for the whole sample and 11% (*COMT*) and 10.5% (*MAOA*) for the analyses performed solely on the Brazilian subsample.

The most recent meta-analysis evaluated about 810 OCD cases and found a positive association between *COMT* rs4680 and OCD in males [41]. These analyses had a power of 49.9% for the total sample and 30.75% for the male sample. As mentioned previously, about 1800 OCD trios were needed to achieve 80% statistical power in a *COMT* rs4680 and OCD association study [40].

The same meta-analysis evaluated about 452 OCD probands regarding the *MAOA* EcoRV polymorphism and found a significant association between T allele and OCD in males [41]. This meta-analysis reached 17.2% statistical power for the *MAOA* polymorphism. The sample number needed to reach 80% power would be more than 2,000 trios [40].

Therefore, the accumulated empirical data to date on the association of *COMT* and *MAOA* polymorphisms and OCD are not enough to refute the null hypothesis and thus, additional association studies are needed to add power in future meta-analyses.

Another limitation was the number of tests performed in this study (more than 120 tests). The multitesting could increase the odds of false positive results. None of the results remained significant after Bonferroni`s correction or permutation analysis. Other investigators, who also had small sample studies, have reported associations between *COMT* and other psychiatric conditions, such as schizophrenia [42,43,44,45,46,47,48,49,50], bipolar disorder [47,50,51]; alcoholism [52,53], substance use disorders [54,55], depression [56,57], and anorexia nervosa [58]. Likewise, *MAO-A* has been associated with attention deficit hyperactivity disorder [59], anxiety disorders [60], major depressive disorder [61], and other psychiatric conditions [9,62,63]. Besides the odds of some false-positive associations, the non-specificity of the phenotypes associated with the *COMT* and *MAO-A* genes might be related to their metabolic functions. Because the *COMT* and *MAO-A* enzymes metabolize a number of important neurotransmitters in the limbic pathways, it is possible that, from a phenomenological perspective, impairment of their function could lead to various psychological presentations. Besides, underlying psychopathology of correlated mental disorders are hypothesized to be dimensional and continuous and to share genetic etiologic factors [64].

Additionally, the genetic heterogeneity of the sample could be another limitation, since the Brazilian population is an admixture of individuals of European, African and indigenous descent. However, the use of a family-based approach in conjunction with the `parental discordance test` minimized the odds of population stratification bias.

In conclusion, the present work investigated eight SNPs from *COMT* gene, five from *MAO-A* gene and the interaction between these two genes in 783 OCD trios using transmission disequilibrium linkage analyses. Analyses using three phenotype variations (from narrow to broad definitions of OCD phenotypes), showed only no significant association. Despite the lack of association, it appears that the broadly-defined OCD, as well as the OCD spectrum disorders, may be interesting phenotypes to be studied in future association analysis. Further genetic association studies with larger samples involving a more comprehensive coverage of polymorphisms in the *COMT* and *MAO-A* genes, as well as other meta-analyses, are needed in order to clarify the biological effect of those genes over the OCD susceptibility phenotypes.

## Supporting Information

S1 Table Association of OCD plus tic disorders with catechol-O-methyltransferase and monoamine oxidase-A single-nucleotide polymorphisms. Legend: SNP: single-nucleotide polymorphism; TDT: transmission/disequilibrium test; OR: TDT odds ratio; CHISQ: TDT chi-square value; P: TDT p value; CHISQ_PAR: parental discordance test chi-square value; P_PAR: parental discordance test p value; CHISQ_COM: combined TDT and parental discordance test chi-square value; P_COM: combined TDT and parental discordance test p value; COMT: catechol-O-methyltransferase; MAO-A: monoamine oxidase-A; NA: not applicable, not enough sample to perform the analysis.
(DOCX)

S2 Table Association of the broadly-defined Obsessive Compulsive Disorder phenotype with catechol-O-methyltransferase and monoamine oxidase-A single-nucleotide polymorphisms. Legend: SNP: single-nucleotide polymorphism; TDT: transmission/disequilibrium test; OR: TDT odds ratio; CHISQ: TDT chi-square value; P: TDT p value; CHISQ_PAR: parental discordance test chi-square value; P_PAR: parental discordance test p value; CHISQ_COM: combined test chi-square value; P_COM: combined test p value; COMT: catechol-O-methyltransferase; MAO-A: monoamine oxidase-A.
(DOCX)

S3 Table Association between obsessive-compulsive spectrum disorders and catechol-O-methyltransferase and monoamine oxidase-A single-nucleotide polymorphisms. Legend: SNP: single-nucleotide polymorphism; TDT: transmission/disequilibrium test; OR: TDT odds ratio; CHISQ: TDT chi-square value; P: TDT p value; CHISQ_PAR: parental discordance test chi-square value; P_PAR: parental discordance test p value; CHISQ_COM: combined test chi-square value; P_COM: combined test p value; COMT: catechol-O-methyltransferase; MAO-A: monoamine oxidase-A
(DOCX)

S4 Table Full dataset used in the analysis. Legend: FAMILY ID: Given code for each family; SUBJECT ID: given code for each subject; FATHER: subject’s father code, 0 if it is not included in the sample; MOTHER: subject’s mother code, 0 if it is not included in the sample; GENDER: subject’s gender = 1 if male, and = 2 if female; DSM OCD: 1 = did not fulfill obsessive-compulsive disorder criteria according the Diagnostic and Statistical Manual for Mental Disorders – forth version, and 2 = did fulfill those criteria; BroadOCD 1 = did not have at least 75% of obsessive-compulsive disorder criteria according the Diagnostic and Statistical Manual for Mental Disorders – forth version, and 2 = did have at least 75% of obsessive-compulsive disorder criteria according the Diagnostic and Statistical Manual for Mental Disorders – forth version; OCD+TIC: 1 = did not fulfill obsessive-compulsive disorder criteria plus Tourette’s syndrome or chronic tic disorder criteria according the Diagnostic and Statistical Manual for Mental Disorders – forth version, and 2 = did fulfill both obsessive-compulsive disorder criteria and Tourette’s syndrome or chronic tic disorder criteria according the Diagnostic and Statistical Manual for Mental Disorders – forth version; SpecOCD: 1 = did not fulfill any of the called Obsessive-compulsive spectrum disorders, which includes broad obsessive-compulsive diagnosis as well as the anxiety disorders, body dysmorphic disorder, and pathological grooming disorders (skin picking and trichotillomania) according the Diagnostic and Statistical Manual for Mental Disorders – forth version.
(XLSX)
