# Supplementary material for: COMT and MAO-A Polymorphisms and Obsessive-Compulsive Disorder: A Family-Based Association Study
Source: PLoS One. 2015 Mar 20;10(3):e0119592. doi: 10.1371/journal.pone.0119592 (PMC4368617; doi:10.1371/journal.pone.0119592)
Supplement: S1 Table — Legend: SNP: single-nucleotide polymorphism; TDT: transmission/disequilibrium test; OR: TDT odds ratio; CHISQ: TDT chi-square value; P: TDT p value; CHISQ_PAR: parental discordance test chi-square value; P_PAR: parental discordance test p value; CHISQ_COM: combined TDT and parental discordance test chi-square value; P_COM: combined TDT and parental discordance test p value; COMT: catechol-O-methyltransferase; MAO-A: monoamine oxidase-A; NA: not applicable, not enough sample to perform the analysis. (DOCX) [file pone.0119592.s001.docx]

Table S1: Association of OCD plus tic disorders with catechol-*O*-methyltransferase and monoamine oxidase-A single-nucleotide polymorphisms.

| **Gene** | **SNP** | **OR** | **CHISQ** | **P** | **CHISQ_PAR** | **P_PAR** | **CHISQ_COM** | **P_COM** |
| --- | --- | --- | --- | --- | --- | --- | --- | --- |
| ***COMT*** | rs737866 | 1.429 | 0.529 | 0.467 | 1 | 0.317 | 0.889 | 0.347 |
|  | rs933271 | 0.9 | 0.053 | 0.818 | NA | NA | 0.053 | 0.818 |
|  | rs5993883 | 0.4 | 1.286 | 0.257 | 0.333 | 0.564 | 0.4 | 0.527 |
|  | rs740603 | 0.5 | 1.333 | 0.248 | 1 | 0.317 | 0.25 | 0.617 |
|  | rs4680 | 1.375 | 0.474 | 0.491 | 0.333 | 0.564 | 0.182 | 0.67 |
|  | rs4646316 | 0.5714 | 0.818 | 0.366 | 2 | 0.157 | 0.077 | 0.782 |
|  | rs165774 | 2.167 | 2.579 | 0.108 | 2 | 0.157 | 1.19 | 0.275 |
|  | rs9332377 | 0.5 | 1.333 | 0.248 | 2 | 0.157 | 2.571 | 0.109 |
| ***MAO-A*** | rs1465107 | 1 | 0 | 1 | 0 | 1 | 0 | 1 |
|  | rs1465108 | 1.25 | 0.111 | 0.739 | 0 | 1 | 10.053 | 0.818 |
|  | rs6323 | 0.6 | 0.5 | 0.48 | 0 | 1 | 10.222 | 0.637 |
|  | rs979606 | 0.8 | 0.111 | 0.74 | 0 | 1 | 10.053 | 0.818 |
|  | rs979605 | 0.8 | 0.111 | 0.74 | 0.111 | 0.739 | 0 | 1 |

SNP: single-nucleotide polymorphism; TDT: transmission/disequilibrium test; OR: TDT odds ratio; CHISQ: TDT chi-square value; P: TDT p value; CHISQ_PAR: parental discordance test chi-square value; P_PAR: parental discordance test p value; CHISQ_COM: combined TDT and parental discordance test chi-square value; P_COM: combined TDT and parental discordance test p value; *COMT*: catechol-*O*-methyltransferase; *MAO-A*: monoamine oxidase-A; NA: not applicable, not enough sample to perform the analysis.
