# Supplementary material for: COMT and MAO-A Polymorphisms and Obsessive-Compulsive Disorder: A Family-Based Association Study
Source: PLoS One. 2015 Mar 20;10(3):e0119592. doi: 10.1371/journal.pone.0119592 (PMC4368617; doi:10.1371/journal.pone.0119592)
Supplement: S2 Table — Legend: SNP: single-nucleotide polymorphism; TDT: transmission/disequilibrium test; OR: TDT odds ratio; CHISQ: TDT chi-square value; P: TDT p value; CHISQ_PAR: parental discordance test chi-square value; P_PAR: parental discordance test p value; CHISQ_COM: combined test chi-square value; P_COM: combined test p value; COMT: catechol-O-methyltransferase; MAO-A: monoamine oxidase-A. (DOCX) [file pone.0119592.s002.docx]

Table S2: Association of the broadly-defined Obsessive Compulsive Disorder phenotype with catechol-*O*-methyltransferase and monoamine oxidase-A single-nucleotide polymorphisms.

| **Gene** | **SNP** | **OR** | **CHISQ** | **P** | **CHISQ_PAR** | **P_PAR** | **CHISQ_COM** | **P_COM** |
| --- | --- | --- | --- | --- | --- | --- | --- | --- |
| ***COMT*** | rs737866 | 0.8065 | 0.643 | 0.423 | 0 |  | 10.529 | 0.467 |
|  | rs933271 | 1.133 | 0.25 | 0.617 | 0.889 | 0.349 | 0 | 1 |
|  | rs5993883 | 1.095 | 0.091 | 0.763 | 2.333 | 0.127 | 1.246 | 0.264 |
|  | rs740603 | 0.7879 | 0.83 | 0.362 | 3.2 | 0.074 | 0.013 | 0.91 |
|  | rs4680 | 0.9167 | 0.13 | 0.718 | 0.25 | 0.617 | 0.294 | 0.588 |
|  | rs4646316 | 1.526 | 2.083 | 0.149 | 0.4 | 0.527 | 2.483 | 0.115 |
|  | rs165774 | 1.129 | 0.242 | 0.622 | 3.522 | 0.06 | 0.281 | 0.596 |
|  | rs9332377 | 0.6786 | 1.723 | 0.189 | 0.333 | 0.564 | 0.83 | 0.362 |
| ***MAO-A*** | rs1465107 | 1.462 | 1.125 | 0.289 | 1.19 | 0.275 | 2.283 | 0.131 |
|  | rs1465108 | 1.462 | 1.125 | 0.289 | 3.24 | 0.072 | 3.947 | **0.047** |
|  | rs6323 | 1.462 | 1.125 | 0.289 | 0.2 | 0.655 | 1.231 | 0.267 |
|  | rs979606 | 1.462 | 1.125 | 0.289 | 0.2 | 0.655 | 1.231 | 0.267 |
|  | rs979605 | 1.615 | 1.882 | 0.17 | 1.19 | 0.275 | 3.073 | 0.08 |

SNP: single-nucleotide polymorphism; TDT: transmission/disequilibrium test; OR: TDT odds ratio; CHISQ: TDT chi-square value; P: TDT p value; CHISQ_PAR: parental discordance test chi-square value; P_PAR: parental discordance test p value; CHISQ_COM: combined test chi-square value; P_COM: combined test p value; *COMT*: catechol-*O*-methyltransferase; *MAO-A*: monoamine oxidase-A
