# Supplementary material for: COMT and MAO-A Polymorphisms and Obsessive-Compulsive Disorder: A Family-Based Association Study
Source: PLoS One. 2015 Mar 20;10(3):e0119592. doi: 10.1371/journal.pone.0119592 (PMC4368617; doi:10.1371/journal.pone.0119592)
Supplement: S3 Table — Legend: SNP: single-nucleotide polymorphism; TDT: transmission/disequilibrium test; OR: TDT odds ratio; CHISQ: TDT chi-square value; P: TDT p value; CHISQ_PAR: parental discordance test chi-square value; P_PAR: parental discordance test p value; CHISQ_COM: combined test chi-square value; P_COM: combined test p value; COMT: catechol-O-methyltransferase; MAO-A: monoamine oxidase-A (DOCX) [file pone.0119592.s003.docx]

Table S3: Association between obsessive-compulsive spectrum disorders and catechol-*O*-methyltransferase and monoamine oxidase-A single-nucleotide polymorphisms.

| **Gene** | **SNP** | **OR** | **CHISQ** | **P** | **CHISQ_PAR** | **P_PAR** | **CHISQ_COM** | P_COM |
| --- | --- | --- | --- | --- | --- | --- | --- | --- |
| ***COMT*** | rs737866 | 0.8333 | 0.455 | 0.5 | 0.818 | 0.366 | 0.97 | 0.325 |
|  | rs933271 | 1.1 | 0.143 | 0.706 | 0.111 | 0.739 | 0.056 | 0.814 |
|  | rs5993883 | 1.143 | 0.2 | 0.655 | 0.727 | 0.394 | 0.731 | 0.392 |
|  | rs740603 | 0.7576 | 1.103 | 0.294 | 2.579 | 0.103 | 0.013 | 0.909 |
|  | rs4680 | 0.9167 | 0.13 | 0.718 | 0.053 | 0.819 | 0.182 | 0.67 |
|  | rs4646316 | 1.429 | 1.588 | 0.208 | 0.5 | 0.48 | 0.83 | 0.362 |
|  | rs165774 | 1.129 | 0.242 | 0.622 | 1 | 0.317 | 0.011 | 0.916 |
|  | rs9332377 | 0.6429 | 2.174 | 0.14 | 0.25 | 0.617 | 2.323 | 0.128 |
| ***MAO-A*** | rs1465107 | 1.583 | 1.581 | 0.209 | 0.133 | 0.715 | 0.41 | 0.522 |
|  | rs1465108 | 1.583 | 1.581 | 0.209 | 0.133 | 0.715 | 1.328 | 0.249 |
|  | rs6323 | 1.462 | 1.125 | 0.289 | 0.727 | 0.394 | 1.852 | 0.174 |
|  | rs979606 | 1.462 | 1.125 | 0.289 | 0.727 | 0.394 | 1.852 | 0.174 |
|  | rs979605 | 1.615 | 1.882 | 0.17 | 3.24 | 0.072 | 4.898 | **0.027** |

SNP: single-nucleotide polymorphism; TDT: transmission/disequilibrium test; OR: TDT odds ratio; CHISQ: TDT chi-square value; P: TDT p value; CHISQ_PAR: parental discordance test chi-square value; P_PAR: parental discordance test p value; CHISQ_COM: combined test chi-square value; P_COM: combined test p value; *COMT*: catechol-*O*-methyltransferase; *MAO-A*: monoamine oxidase-A
